# Mechanisms and Anatomic Distribution of Hand and Wrist Injuries in the Swedish Hockey League: A 3-Season Registry Study

**DOI:** 10.1177/23259671261486943

**Published:** 2026-10-07

**Authors:** Daniel Muder, Mikael Swarén

**Affiliations:** † Department of Surgical Sciences/Orthopaedics & Hand Surgery, Uppsala University, Uppsala, Sweden; ‡ Center for Clinical Research Dalarna–Uppsala University, Falun, Sweden; § Swedish Unit for Metrology in Sports, Department of Sport, Fitness and Health, Dalarna University, Falun, Sweden; Investigation performed at Center for Clinical Research Dalarna–Uppsala University, Falun, Sweden

**Keywords:** ice hockey, hand injuries, wrist injuries, fractures, epidemiology, sports injury, Swedish Hockey League

## Abstract

**Background:**

Hand and wrist injuries are common in ice hockey and contribute substantially to time loss and performance limitations. However, prospective registry-based data from professional leagues remain limited.

**Purpose:**

To describe the anatomic distribution, mechanisms, and severity of hand, wrist, and forearm injuries in professional ice hockey players across 3 elite seasons.

**Study Design:**

Descriptive epidemiology study.

**Methods:**

Prospectively collected data from a professional ice hockey injury registry were analyzed. Hand, wrist, and forearm injuries sustained during 3 consecutive seasons (2022/2023 to 2024/2025) were included (n = 113). Variables included injury type, mechanism, anatomic location, playing situation, player position, laterality, and time loss. Injury severity was defined according to days lost.

**Results:**

A total of 113 injuries were identified, 85.8% occurring during games. Fractures represented the most common injury type (54.0%) and resulted in significantly greater time loss (median, 32 days) compared with sprains (6 days) and contusions/lacerations (5 days). Puck contact was the most common injury mechanism (45.1%), and fractures accounted for 70.6% of puck-related injuries. Collision-related injuries demonstrated broader anatomic distribution involving the wrist and forearm and were more frequently associated with sprains. Injuries clustered to the hand region across playing positions, with limited positional or laterality-related differences.

**Conclusion:**

Hand, wrist, and forearm injuries in professional ice hockey are predominantly fracture related and commonly caused by puck contact. Injury patterns demonstrated clustering to the distal hand region.

Ice hockey is a high-intensity collision sport characterized by high skating speeds, rapid directional changes, and frequent contact with opponents, boards, sticks, and the puck. These features place players at substantial risk of traumatic injuries across all levels of play.^2,11,15^

Upper body injuries, including the shoulder, account for approximately 22% of all injuries in elite ice hockey.^15^ In elite professional hockey, approximately 16% of reported injuries involve the elbow, forearm, wrist, or hand.^1,16^ These injuries range from minor contusions to fractures and ligament injuries and may result in substantial time loss and reduced player availability.^4,13^

Recent epidemiologic data further emphasize the predominance of upper extremity trauma in ice hockey. A nationwide US study demonstrated that upper extremity and trunk injuries accounted for more than 80% of all fractures, with distal upper extremity structures including the forearm, wrist, hand, and fingers comprising 44.88% of all fractures.^10^ Hand fractures were significantly more common in older players, suggesting an age-dependent shift in injury patterns potentially related to increased puck velocity, collision intensity, and game speed.^10^

Hand and wrist function is essential for stick handling, shooting, and puck control. Even minor injuries may impair technical performance and upper limb function. Grip strength has been associated with athletic performance and upper extremity function, suggesting that hand integrity may influence performance beyond return to play alone.^6,12,14^ Recent National Hockey League (NHL) studies have reported variable postinjury performance outcomes following hand fractures, with evidence of both reduced performance after return to competition and recovery to preinjury performance within 1 year.^4,5^

The mechanisms of hand and wrist injuries in ice hockey are multifactorial. Prior studies have identified collisions, board contact, stick contact, and puck impacts as major injury mechanisms.^8,13^ Puck-related trauma is of particular concern because slap shots may generate forces exceeding experimentally derived fracture thresholds despite modern glove protection.^7^ Phalangeal and metacarpal fractures appear especially common in professional hockey populations and are frequently associated with prolonged absence from play.^5^

Despite the recognized burden of these injuries, much of the existing literature relies on media reports, insurance databases, or emergency department data, limiting injury characterization and sport-specific clinical detail.^4,9^ Prospective registry–based surveillance systems allow more detailed assessment of injury type, mechanism, anatomic distribution, and time loss according to international consensus recommendations.^3^

A prospective professional ice hockey injury registry provides a unique opportunity to study hand, wrist, and forearm injuries using systematically collected elite-level data. However, detailed register-based epidemiologic studies focusing specifically on distal upper extremity injuries in professional hockey remain limited.

Therefore, the aim of this study was to describe the anatomic distribution, mechanisms, and severity of hand, wrist, and forearm injuries in professional male ice hockey players using registry data. Secondary aims were to explore differences related to player position, laterality, and injury type in relation to time loss.

## Methods

### Study Design and Setting

This observational, descriptive, register-based study used data from the prospective injury registry of the Swedish Hockey League. The registry follows International Olympic Committee consensus recommendations for injury surveillance in sport^3^ and has been operational since 2008. Since 2022, a refined prospective data collection system has been implemented in which team medical staff register all injuries.

All hand, wrist, and forearm injuries occurring during the 2022/2023, 2023/2024, and 2024/2025 seasons were included. Both fracture and soft tissue injuries were eligible for inclusion.

Ethical approval was obtained from the Swedish Ethical Review Authority (Dnr 2025-01728-01), and the study was registered at ClinicalTrials.gov (NCT07031869) before data extraction.

### Variables and Data Collection

Recorded variables included injury location (hand, wrist, forearm), injury type, injury mechanism, playing situation (game/training), game timing, player position, laterality, and time loss. Mechanisms were categorized according to the registry, with tackling referring to player-to-player body checking and collision referring to other collision events, including accidental player-to-player contact and contact with the boards. For analyses of hand position on the stick, shooting side and injury laterality were used to classify injuries as involving the upper or lower hand, defined as the hand positioned higher or lower on the stick shaft during play, respectively. Injury severity was categorized according to time loss. Time loss was defined as the number of days from injury until the player was fully available for training and eligible for selection for competition. Sprains comprised ligamentous injuries and distortions, whereas soft tissue injuries involved tendon and muscle injuries; these categories were therefore analyzed separately.

### Statistical Analysis

Data were assessed for normality using the Shapiro-Wilk test. Continuous variables were nonnormally distributed and are presented as median (IQR) and mean ± SD where appropriate. Categorical variables are presented as frequencies and percentages.

Associations between categorical variables were analyzed using Pearson chi-square or Fisher exact tests when assumptions were not met. Differences in time loss between groups were analyzed using Mann-Whitney *U* or Kruskal-Wallis tests. Significant Kruskal-Wallis findings were followed by Dwass-Steel-Critchlow-Flinger post hoc comparisons.

Missing data were reported and not imputed. Because time-loss data were substantially skewed, median values were prioritized for interpretation.

All analyses were performed using SPSS version 30.0 (SPSS, Inc).

## Results

### Cohort and Overall Injury Burden

A total of 113 hand, wrist, and forearm injuries were recorded in professional male ice hockey players (mean age, 25 ± 9 years). Details on seasonal distribution and playing situation are presented in Table 1.

**Table 1:** Seasonal Distribution and Playing Context of Hand, Wrist, and Forearm Injuries in 3 Seasons (2022/2023 to 2024/2025)*^*a*^*

Variable	Category	No. (%)
Season	2022/2023	44 (38.9)
	2023/2024	34 (30.1)
	2024/2025	35 (31.0)
Playing situation	Game	97 (85.8)
	Training	16 (14.2)

a No inferential statistics were performed.

### Anatomic Distribution of Injuries

Hand injuries represented the most common anatomic location across all player positions. Among goalkeepers, all injuries involved the hand, compared with 57% in defensemen and 59% in forward. Wrist injuries accounted for approximately one-fourth of injuries in defensemen and forward, whereas forearm injuries were less common.

The anatomic distribution demonstrated clear clustering to the hand region across all player positions (Figure 1).

**Figure 1.**
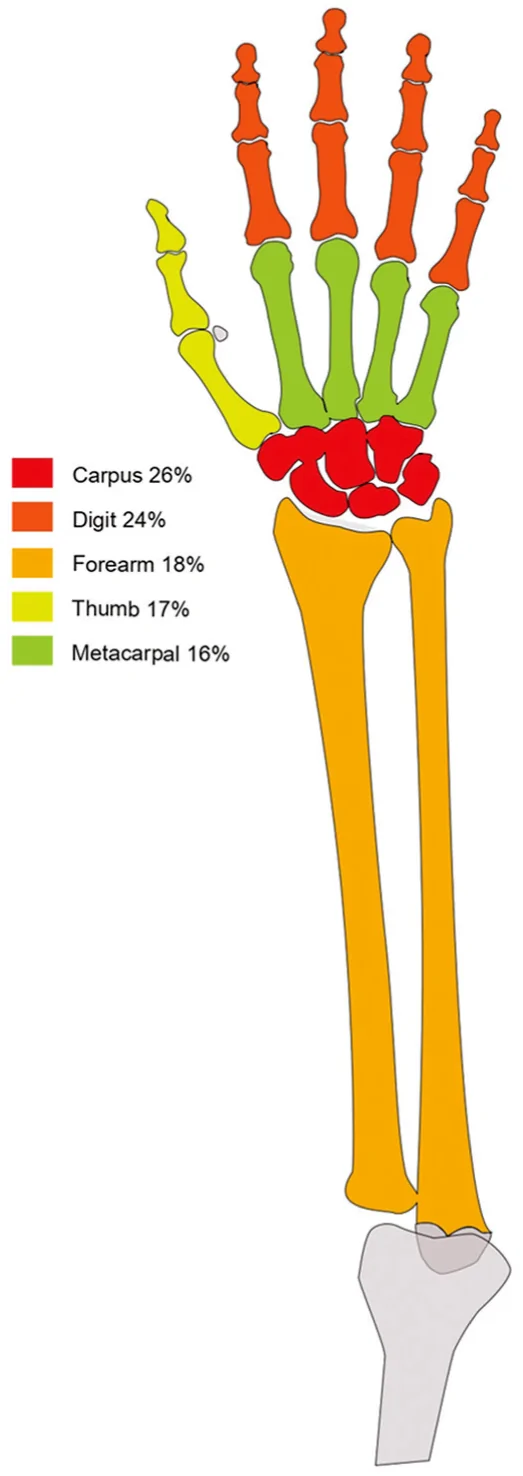
Anatomic distribution of hand, wrist, and forearm injuries in professional ice hockey players.

### Injury Type and Severity Profile

Fractures were the most common injury type, accounting for 54.0% of all injuries (n = 61), followed by sprains (27.4%) and contusions/lacerations (15.0%). Among the 61 fractures, 20 (32.8%) involved the phalanges, 19 (31.1%) the metacarpals, 12 (19.7%) the forearm, and 4 (6.6%) the carpus; 6 (9.8%) involved the thumb without further anatomic specification. Median time loss for the overall cohort was 16 days (IQR, 39 days), although the distribution was highly skewed because of a limited number of prolonged absences. For sprains, the median time loss was 6 days (IQR, 22 days), with 2 prolonged absences of 461 and 811 days contributing substantially to the large standard deviation.

Time loss differed significantly between injury types (*P* < .001), with fractures resulting in a significantly longer absence than both sprains and contusions/lacerations. Fractures also accounted for most moderate and severe injuries (Table 2).

**Table 2:** Injury Type and Time Loss Among Hand, Wrist, and Forearm Injuries in Professional Ice Hockey*^*a*^*

Injury Type	No. (%)	Time Loss, Median (IQR), d	Mean ± SD, d
Fracture	61 (54.0)	32 (35)	41.0 ± 56.9
Sprain (ligamentous/distortion)	31 (27.4)	6 (22)	52.0 ± 162.9
Contusion/laceration	17 (15.0)	5 (9)	7.5 ± 7.6
Soft tissue injury (tendon/muscle)	1 (0.9)	—	—
Rare injuries (dislocation/unspecified)	3 (2.7)	—	—
Total cohort	113 (100)	16 (39)	37.8 ± 95.3

a Time loss differed significantly between injury types (Kruskal-Wallis, *P* < .001). Post hoc pairwise comparisons (Dwass-Steel-Critchlow-Flinger) showed significantly longer time loss for fractures compared with sprains and contusions/lacerations (adjusted *P* = .001 for both comparisons). Rare and soft tissue injuries are descriptive only due to low numbers.

The distribution of injury types across anatomic regions is illustrated in Figure 2.

**Figure 2.**
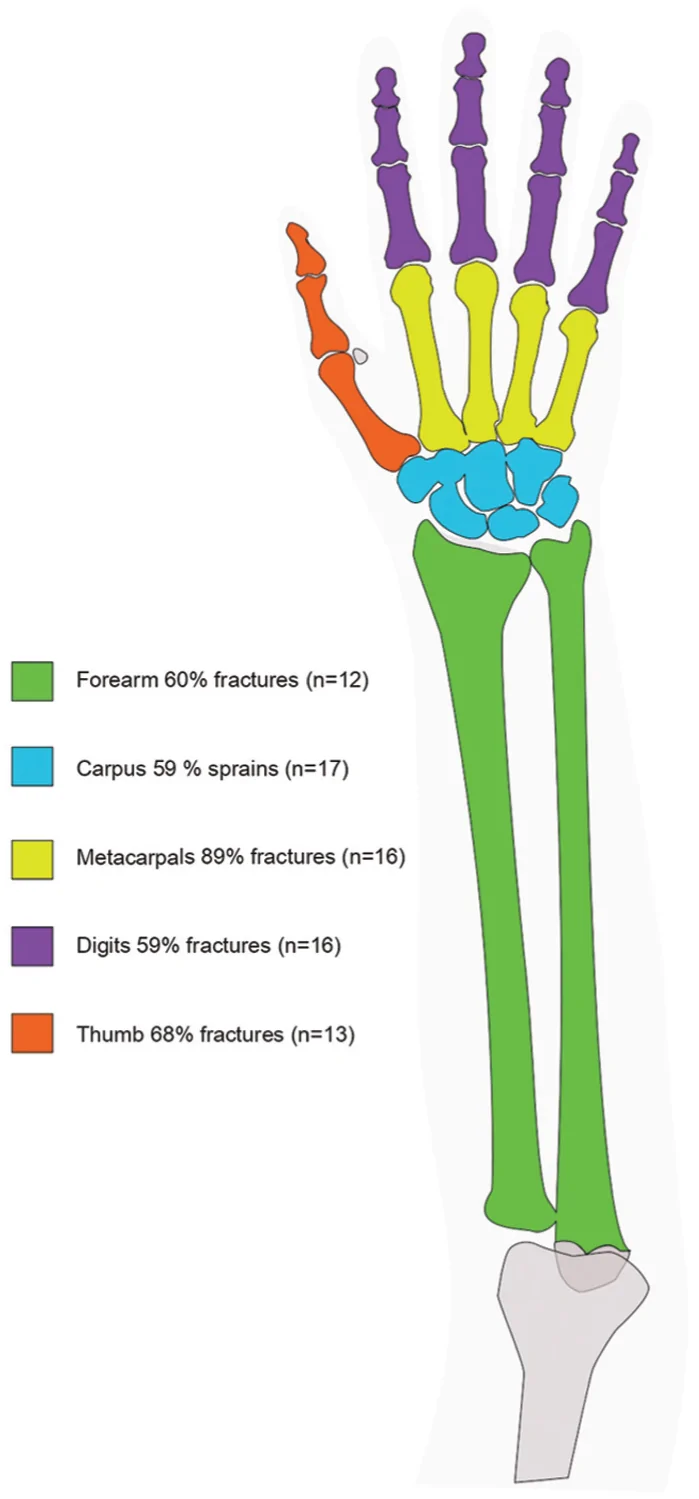
Distribution of injury types across anatomic regions of hand, wrist, and forearm injuries in professional ice hockey players.

### Injury Mechanisms

Puck contact was the most common injury mechanism, accounting for 45.1% of injuries, followed by collision-related trauma (25.7%), tackling (11.5%), and stick contact (8.0%).

Fractures were particularly common following puck contact, accounting for 70.6% of puck-related injuries. In contrast, collision-related injuries demonstrated a broader distribution between fractures and sprains. Mechanism-specific injury distributions are presented in Table 3.

**Table 3:** Mechanism-Specific Distribution of Injury Types in Hand, Wrist, and Forearm Injuries in Professional Ice Hockey*^*a*^*

Mechanism	Fracture	Sprain (Ligamentous/Distortion)	Soft Tissue	Contusion/Laceration	Rare	Total
Puck contact	36 (70.6)	5 (9.8)	0 (0.0)	10 (19.6)	0 (0.0)	51 (100)
Stick contact	4 (44.4)	2 (22.2)	1 (11.1)	2 (22.2)	0 (0.0)	9 (100)
Tackling	8 (61.5)	5 (38.5)	0 (0.0)	0 (0.0)	0 (0.0)	13 (100)
Fall	3 (60.0)	2 (40.0)	0 (0.0)	0 (0.0)	0 (0.0)	5 (100)
Collision (board/player)	10 (34.5)	12 (41.4)	0 (0.0)	5 (17.2)	2 (6.9)	29 (100)
Skate contact	0 (0.0)	4 (100.0)	0 (0.0)	0 (0.0)	0 (0.0)	4 (100)
Other	0 (0.0)	0 (0.0)	0 (0.0)	0 (0.0)	1 (100.0)	1 (100)
Missing	0 (0.0)	1 (100.0)	0 (0.0)	0 (0.0)	0 (0.0)	1 (100)
Total	61 (54.0)	31 (27.4)	1 (0.9)	17 (15.0)	3 (2.7)	113 (100)

a Values are presented as number (%) within each mechanism category. Percentages in the total row represent percentages of the overall cohort. No inferential statistics were performed.

Puck-related trauma predominantly involved the phalanges and metacarpals, whereas collision-related injuries more commonly affected the wrist and forearm.

### Game Context and Timing

Most injuries occurred during games (85.8%) and the regular season (86.7%). A chi-square goodness-of-fit test demonstrated a nonuniform distribution of injuries across regulation play segments (χ^2^ = 29.43, *df* = 2, *P* < .001), with a higher-than-expected proportion of injuries occurring during the second segment of play (mid-period phase) and fewer injuries in the first segment.

### Player Position and Laterality

Forward accounted for 55.8% of injuries, followed by defensemen (38.9%) and goalkeepers (3.5%). Fractures represented the dominant injury type across all playing positions.

No clinically meaningful differences in overall injury distribution or severity were observed between playing positions. In contrast, a more nuanced pattern was observed between injuries to the upper and lower hands on the stick, with similar overall distributions but subtle differences in injury type and mechanism.

Among upper hand injuries (n = 56), fractures accounted for 62.5% (n = 35) and contusions/lacerations for 5.4% (n = 3), compared with 48.1% (n = 26) and 22.2% (n = 12), respectively, among lower hand injuries (n = 54). Puck/stick-related mechanisms accounted for 58.9% (n = 33) of upper hand and 46.3% (n = 25) of lower hand injuries, whereas tackling/collision-related mechanisms accounted for 26.8% (n = 15) and 48.1% (n = 26), respectively.

### Exploratory Findings

Exploratory analyses showed that radial-sided injuries involving the thumb, index finger, and radial metacarpals accounted for 62.5% (40/64) of hand injuries classified as radial, central, or ulnar. Among game injuries, 55.7% (54/97) occurred in the defensive zone, and 62.5% (25/40) of radial-sided injuries were puck related.

These exploratory findings should be interpreted as hypothesis-generating because of the small subgroup sizes and should not be considered evidence of definitive anatomic or mechanism-specific injury patterns.

## Discussion

The principal findings of this nationwide register-based study were that fractures represented the dominant injury type and accounted for the greatest time loss, puck contact was the most common injury mechanism and was strongly associated with distal hand fractures, and injuries consistently clustered to the hand region regardless of player position.

The present findings reinforce the substantial burden of distal upper extremity trauma in elite ice hockey. Hand injuries predominated across all player positions, including exclusive hand involvement among goalkeepers, reflecting continuous exposure during puck handling, shooting, shot blocking, and defensive play.

These findings align with recent epidemiologic data demonstrating that distal upper extremity injuries account for a substantial proportion of hockey-related fractures.^10^ Similar upper extremity predominance has been reported in international tournament surveillance studies and National Collegiate Athletic Association cohorts.^15^ However, unlike several previous studies based on emergency department databases or publicly available injury reports, the present study provides prospective league-wide medical registry data with detailed characterization of injury mechanisms, anatomic distribution, and time loss.

The clustering of injuries to the distal hand region suggests that injury risk is concentrated to structures exposed during high-velocity puck and stick interactions. From both clinical and performance perspectives, these injuries are highly relevant because even relatively minor impairments may affect grip strength, stick control, and shooting performance. Recent NHL studies have reported variable postinjury performance outcomes, with reductions in selected performance measures after return to competition but recovery to preinjury performance within 1 year in another cohort.^4,5^

Fractures accounted for more than half of all injuries and were associated with substantially greater time loss than soft tissue injuries, reinforcing fractures as the primary contributor to injury burden in this cohort. Metacarpal and phalangeal fractures appear particularly important in elite hockey because of both their frequency and their functional consequences.^5,10^

Puck contact was the dominant injury mechanism and demonstrated a strong association with distal hand fractures involving the metacarpals and phalanges. These findings are consistent with biomechanical studies demonstrating that puck impacts may exceed fracture thresholds despite modern glove protection.^7^ Similar mechanisms have been reported in elite hockey cohorts in which blocked shots and puck contact represent major contributors to hand trauma.^5,8^

In contrast, collision-related injuries demonstrated a broader anatomic distribution involving the wrist and forearm and included a greater proportion of sprains. Prior studies have similarly identified player-to-player collisions and board contact as important contributors to upper extremity trauma in hockey.^13^ Together, these findings suggest partially distinct injury pathways related to puck impact and collision exposure.

Exploratory analyses further suggested a predominance of radial-sided injuries involving the thumb, index finger, and radial metacarpals, particularly during puck-related trauma and defensive-zone play. Although hypothesis-generating, this pattern may represent a clinically relevant exposure pathway associated with shot blocking and defensive play.

Most injuries occurred during games, with a nonuniform distribution across regulation play segments and a higher proportion of injuries occurring during the second segment of play. A similar predominance of injuries during the second period has been reported in international ice hockey.^15^

No clinically meaningful differences in injury severity or overall injury distribution were observed between player positions or between upper and lower hand injuries. These findings suggest that injury risk may be more strongly associated with situational exposure than with player role alone. However, recent NHL data demonstrated a greater prevalence of fractures involving the nondominant shooting hand, indicating that stick mechanics and hand positioning may influence exposure patterns in elite hockey players.^5^

The primary strength of this study is the use of a prospective nationwide injury registry with standardized league-wide injury surveillance according to International Olympic Committee recommendations. Inclusion of multiple professional seasons improves the robustness and generalizability of the findings within elite professional hockey. In addition, the registry-based design allowed detailed characterization of injury type, anatomic location, mechanism, and time loss.

Several limitations should be acknowledged. First, subgroup analyses were based on relatively small numbers, limiting statistical power in certain comparisons. Second, time-loss data were substantially skewed because of a limited number of prolonged absences. Third, athlete exposure data were unavailable, preventing calculation of exposure-adjusted incidence rates and relative injury risk. Detailed information regarding treatment, specific ligament involvement, and nerve, vascular, or tendon involvement in lacerations was not systematically available in the registry, limiting further characterization of these injuries. Finally, the exploratory analyses regarding radial-sided injury patterns and defensive-zone exposure should be interpreted cautiously and considered hypothesis-generating.

## Conclusion

Hand, wrist, and forearm injuries in elite ice hockey are dominated by fractures and puck-related trauma, with consistent clustering to the distal hand region and substantial variability in time loss. Distinct injury patterns appear to exist between puck-related and collision-related trauma, whereas positional and laterality effects appear limited. These findings provide a detailed epidemiologic framework that may inform future injury prevention strategies and protective equipment development in elite ice hockey.
